# Deep neural networks effectively model neural adaptation to changing background noise and suggest nonlinear noise filtering methods in auditory cortex

**DOI:** 10.1016/j.neuroimage.2022.119819

**Published:** 2022-12-16

**Authors:** Gavin Mischler, Menoua Keshishian, Stephan Bickel, Ashesh D. Mehta, Nima Mesgarani

**Affiliations:** a Mortimer B. Zuckerman Mind Brain Behavior, Columbia University, New York, United States; b Department of Electrical Engineering, Columbia University, New York, United States; c Hofstra Northwell School of Medicine, Manhasset, New York, United States

**Keywords:** Adaptation, Auditory neuroscience, Deep neural networks, Modeling

## Abstract

The human auditory system displays a robust capacity to adapt to sudden changes in background noise, allowing for continuous speech comprehension despite changes in background environments. However, despite comprehensive studies characterizing this ability, the computations that underly this process are not well understood The first step towards understanding a complex system is to propose a suitable model, but the classical and easily interpreted model for the auditory system, the spectro-temporal receptive field (STRF), cannot match the nonlinear neural dynamics involved in noise adaptation. Here, we utilize a deep neural network (DNN) to mode neural adaptation to noise, illustrating its effectiveness at reproducing the complex dynamics at the levels of both individual electrodes and the cortical population. By closely inspecting the model’s STRF-like computations over time, we find that the model alters both the gain and shape of its receptive field when adapting to a sudden noise change. We show that the DNN model’s gain changes allow it to perform adaptive gain control, while the spectro-temporal change creates noise filtering by altering the inhibitory region of the model’s receptive field Further, we find that models of electrodes in nonprimary auditory cortex also exhibit noise filtering changes in their excitatory regions, suggesting differences in noise filtering mechanisms along the cortical hierarchy. These findings demonstrate the capability of deep neural networks to model complex neural adaptation and offer new hypotheses about the computations the auditory cortex performs to enable noise-robust speech perception in real-world, dynamic environments.

## 1. Introduction

Humans have a remarkable ability to understand speech despite the many sources of background noise that are constantly present in real-world environments. In complex acoustic scenes, the statistics of the background noise may suddenly change, such as when a speaker and listener walk from a busy street into a restaurant which requires rapid adaptation to varying noise properties. Studies have identified noise-robust representations of sound in the auditory cortex of humans (Ding and Simon, 2013 ; Kell and McDermott, 2019 ; Kell and McDermott, 2017) and model animals (Mesgarani et al., 2014; Moore et al., 2013; Narayan et al., 2007; Rabinowitz et al., 2013; Schneider and Woolley, 2013), as well as in subcortical regions (Dean et al., 2005; Finlayson and Adam, 1997; Ingham and McAlpine, 2004 ; Wen et al., 2009). Recently, intracranial recording in humans showed that neural sites in the auditory cortex exhibit rapid adaptation in response to sudden changes in background noise, which allows them to recover the momentarily disturbed speech features (Khalighinejad et al., 2019). However, the computational mechanism which enables this adaptation is still not well understood.

Adaptation to sensory context is a critical ability of sensory neurons to optimally encode sensory inputs in a dynamic environment (Fairhall et al., 2001; Ulanovsky et al., 2004). Past research has identified adaptive gain control mechanisms, including adaptation to the spectro-temporal contrast (Cooke et al., 2018; Rabinowitz et al., 2011; Willmore et al., 2014), dynamic range (Herrmann et al., 2014; Wen et al., 2009, 2012), and intensity (Watkins and Barbour, 2008) of an auditory stimulus. These mechanisms have been found to facilitate adaptation at a small scale to synthetic stimuli, permitting a more efficient and consistent encoding of varying inputs (David, 2018; Lohse et al., 2020). However, given the complexity of real-world auditory environments where listeners attend to speech while background noises vary, it is still unclear if simple mechanisms such as adaptive gain control are enough to fully explain auditory cortical adaptation, or how it mani-fests alongside other adaptive computations. Understanding the overall filtering being performed to adapt to sudden noise changes will provide useful insights into the capacity of noise-robust speech representations in the human auditory cortex.

Rather than directly searching for such an all-encompassing mechanism, we took a data-driven modeling approach to learn and understand nonlinear transformations. The classical model for the auditory cortex is the spectro-temporal receptive field (STRF) (Aertsen et al., 1981; Klein et al., 2006; Theunissen et al., 2000), which uses a linear transformation to predict neural responses from spectro-temporal input. As a linear model, the STRF is easily inspected and understood. However, it is severely underpowered in modeling complex dynamics (Keshishian et al., 2020), such as the nonlinear adaptation that arises from sudden noise changes. Because of this, many attempts have been made to extend the STRF to model adaptation (see David, 2018 for a review). One of the most common is the linear-nonlinear (LN) STRF, which includes a static nonlinearity, such as a sigmoid, after the linear STRF is applied, inspired by the nonlinear activation thresholds of neurons (Calabrese et al., 2011; David et al., 2009). Others have added a gain normalization mechanism to a STRF model to allow it to deal with changing spectro-temporal contrast (Rabinowitz et al., 2012). The recent short-term plasticity (STP) model also incorporates short-term depression into the linear model, whereby stimulation of the model causes a momentary decrease in its output strength for subsequent stimuli (David et al., 2009; David and Shamma, 2013; Espejo et al., 2019). All of these models incorporate specific changes or additions to the STRF formulation which allow them to better predict neural responses. However, they are typically more difficult to interpret than a linear STRF (Keshishian et al., 2020), and each extension’s parameterization was designed to allow the model to fit a specific type of response pattern, embedding a bias in the model in the form of the neural responses that it was designed to mimic. Thus, our understanding of the complex computations that give rise to auditory cortical adaptation is still incomplete since no model has been proposed which could explain a wide array of adaptation properties simultaneously, a prerequisite for any model generalizing to real-world acoustic conditions.

An alternative data-driven modeling framework that can alleviate the limitations of previous neural adaptation models is a deep neural network (DNN). These models have a high capacity to learn complex nonlinear transformations directly from the data without the need to speculate the exact type of nonlinearities that occur in neural adaptation. They have also been used to study a wide variety of neural systems in the auditory cortex, from highly specialized architectures that simulate firing patterns of individual neurons (Kudela et al., 2018) to general architectures that model the auditory cortical hierarchy (Kell et al., 2018). When used as auditory encoding models, DNNs have been able to consistently outperform other linear or nonlinear encoding models while capturing a wide set of computations throughout the auditory cortex (Keshishian et al., 2020; Pennington and David, 2022). It has been shown that the computations of a certain class of DNN can be visualized at each point in time as a dynamic STRF (dSTRF) (Keshishian et al., 2020), reducing the complexity of analysis that typically comes with nonlinear encoding models.

In this work, we investigated the use of DNNs to model auditory cortical responses to speech in noise and adaptation to sudden noise changes. We trained DNN models to predict the neural responses of neurosurgical patients implanted with depth and surface intracranial electrodes (iEEG) who listened to speech in the presence of changing background noise, a task which requires a high degree of nonlinear adaptation (Khalighinejad et al., 2019). We first show that DNNs significantly outperform linear STRF and STP models at predicting neural responses in individual electrodes in modeling neural adaptation. Furthermore, the models are still highly interpretable through their dSTRFs, and we identify noise-dependent gain and spectro-temporal changes in their filtering immediately following noise changes. We show that these dynamics are related to well-studied neural mechanisms of noise adaptation, and we provide evidence that these dynamics are involved in the DNN’s improved modeling of nonlinear adaptation. Furthermore, we identify two classes of electrodes separated by neural response properties and anatomical location whose models show distinct adaptive dynamics. These modeling results present promising directions for the identification of the precise computations underlying noise-robust encoding in the human auditory cortex.

## 2. Results

**Fig. 1.**
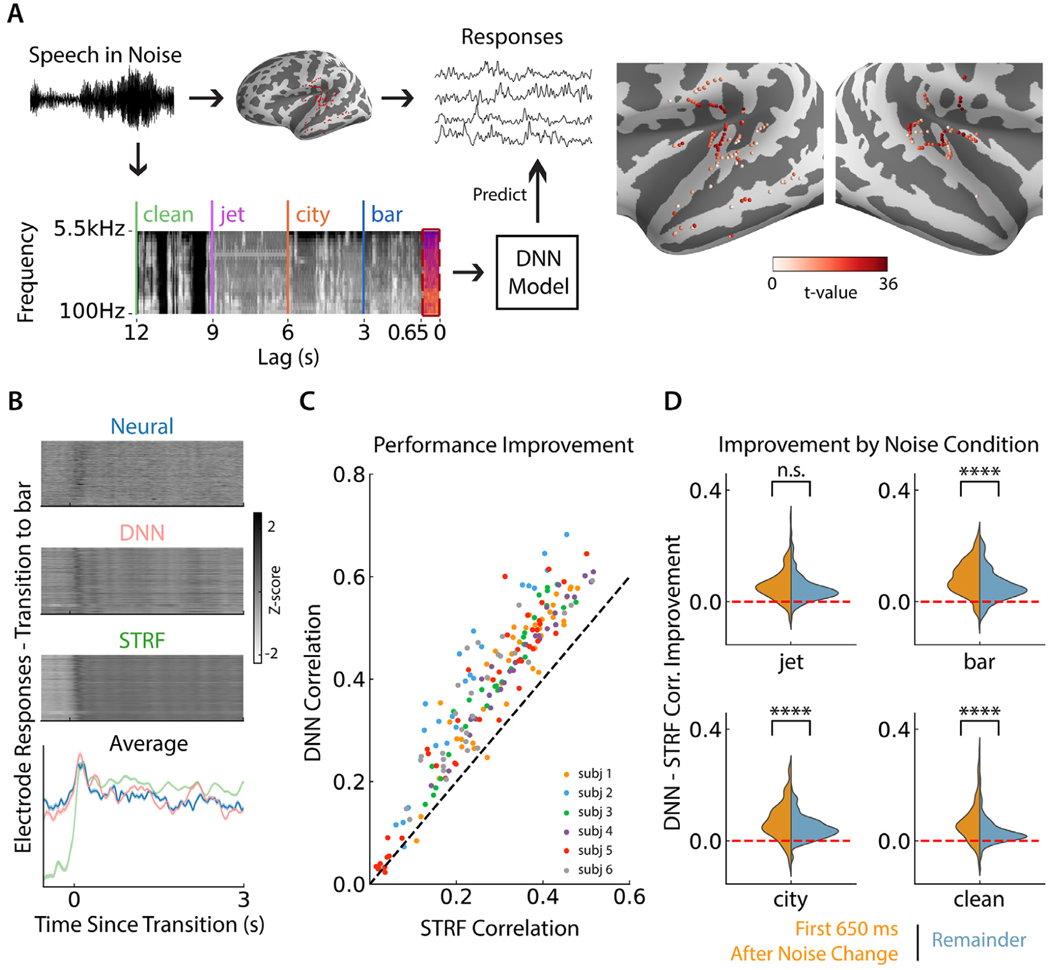
DNN modeling paradigm and performance improvement. (A) Illustration of the DNN modeling paradigm. Speech embedded in background noise which regularly changed was played to subjects while iEEG was recorded. The time-frequency representation of the stimulus was fed to a DNN model with a receptive field of the past 650 ms to predict each electrode’s neural response. T-value for responsive electrodes in both hemispheres. (B) Neural responses of each electrode, and those predicted by the DNN and STRF models, averaged over all transitions to bar noise. The bottom plot shows the average of each of these three models over electrodes. Responses are z-scored for the purposes of maintaining a consistent color scale and range for this figure. (C) Predicted response correlation of each electrode by the DNN compared to the STRF over the full task, colored by subject identity. (D) Correlation improvement of the DNN over the STRF, computed in each noise condition individually. For each noise condition, improvement is further divided into the time period during adaptation, which is the first 650 ms after any noise change, and the remainder of each noise condition. Stars indicate significance level from a subject-controlled paired *t*-test showing greater improvement in the adaptation period than the remainder. (*p < 0.05, **p < 0.01, ***p < 0.001, ****p < 0.0001).

We recorded iEEG from 6 subjects (native speakers of American English) who were undergoing clinical evaluation before epilepsy surgery. Electrode coverage varied by subject according to clinical placement, but only speech responsive electrodes were kept for analysis, as determined by a paired *t*-test between each electrode’s response to speech vs silence (FDR corrected (Holm, 1979), *p* < 0.01), depicted in Fig. 1 A. These electrodes were located in Heschl’s gyrus (HG), superior temporal gyrus (STG), transverse temporal sulcus, planum temporale, and middle temporal gyrus (MTG). Subjects listened to continuous speech from male and female speakers reading a story in which the background noise changed every 3 or 6 s between bar noise, city noise, jet noise or no noise (clean speech), creating a large set of 3/6 s windows of stimuli and transition-aligned neural responses. These three noise types were used because they sample a diverse range of frequency content, stationarity, and speech similarity (Khalighinejad et al., 2019), potentially requiring a model to operate differently in each noise case. Additional description of the stimulus design and rationale can be found in a previous work (Khalighinejad et al., 2019). To make sure subjects were focused on the task, the stimulus was paused at random points throughout the task and the subject was asked to repeat the last sentence they heard. All subjects were engaged in the task and could repeat the most recent sentences. Here we define the neural responses as the envelope of the high-gamma band (70–150 Hz) of the neural recordings.

We then trained both STRF and DNN models to predict the neural responses from the stimulus spectrogram at a sampling rate of 100 Hz. In order to identify robust properties of the DNN models, they were trained in a cross-validated jackknifing procedure where multiple models were trained using different portions of the training data to predict the same withheld test data. The DNN model was a convolutional neural network (CNN) with a receptive field containing the last 650 ms window of the stimulus, illustrated in Fig. 1 A. This window size was chosen to give the model sufficient ability to reproduce the adaptation effects which can last as long as 700 ms for some electrodes (Khalighinejad et al., 2019), while allowing for a simple model architecture with fixed kernel size (see Materials and methods). Longer receptive fields and different model architectures had no significant effect on model performance, as shown in Fig. S1. To provide a fair comparison, the STRF models were trained and tested in the same manner as the DNN models.

### 2.1. DNN outperforms linear STRF and STP in adaptation modeling

We first sought to confirm that the DNN was a sufficiently good model of neural adaptation by comparing the neural response predictions by each class of model. As seen in Fig. 1B, predicted responses around noise changes are qualitatively much better from DNNs than from STRFs, maintaining the baseline response level and tracking the neural response very well, which the STRF does not achieve. We computed the correlation between each model’s predictions and the true neural response over the full task and found that the DNN significantly outperformed the STRF (subject-controlled paired *t*-test, *p* < 0.001), as shown in Fig. 1C, with a median improvement in correlation of 0.095. Next, we wanted to ensure that this improvement in correlation was not primarily due to improvements in the predictions after adaptation had taken effect, but that the model was doing significantly better than the STRF during the critical adaptation period that we wished to study. Therefore, we computed the correlation for each electrode and noise type for two different sections of the response: the adaptation period of the first 650 ms after a noise transition, where the noise type changes inside the model’s receptive field, and the remaining time where the noise type is constant within the model’s receptive field. As shown in Fig. 1D, the DNN demonstrated a greater improvement over the STRF during the adaptation period compared to afterward for bar, city, and clean conditions (subject-controlled paired *t*-test, all *p* < 0.001). We also compared the DNN’s performance to that of a short-term plasticity model (STP) (David et al., 2009; David and Shamma, 2013; Espejo et al., 2019). The DNN achieved a significant correlation improvement over the STP model as well (subject-controlled *t*-test, *p* < 0.001), which is shown in supplemental Fig. S2A. As we did for the STRF comparison, we also divided the correlations between the first 650 ms and the remainder after transitions. Supplemental Fig. S2B shows the distributions of these correlation improvements. We found that the DNN performed significantly better than the STP model for both stimulus periods (subject-controlled paired *t*-test, *p* < 0.001 for the first 650 ms and *p* < 0.001 for the remainder), but there was no significant difference in improvement between the during- and after-adaptation periods (subject-controlled paired *t* -test, *p* > 0.05). Additionally, a separate STRF trained in each noise condition did not improve performance compared to the baseline STRF model, and in fact performed slightly worse on average (subject-controlled paired *t*-test, *p* < 0.05) and performed significantly worse than the DNN model in all noise conditions (subject-controlled paired *t* -test, *p* < 0.001).

### 2.2. DNN is interpretable through its dSTRF which adapts to noise changes

Having confirmed that the DNN was performing well in predicting neural adaptation, we next studied the DNN model’s computations to understand how it achieved its high performance. To do this, we extracted the model’s dSTRF over the course of the stimulus. The dSTRF is a DNN’s equivalent piecewise linear model which allows us to interpret the DNN’s operation at each instant as a spectro-temporal filter similar to a STRF (Keshishian et al., 2020). In a feedforward neural network with rectified linear unit activations (ReLU), for a given stimulus input, certain nodes will be active, and thus can be replaced by a unity function, while any inactive nodes can be removed. This is illustrated in supplemental Fig. S3. Then, the remaining nodes can be multiplied, which entails the multiplication of a series of linear weights, leaving a single linear equivalent to the entire network for this input instance (see Materials and methods for more details). For a given stimulus input, the dSTRF weights perform the exact same computation as the DNN model, and the full DNN can be thought of as selecting which linear filter to use depending on the input it is given. The dSTRF can be visualized in the same frequency-by-lag manner as a STRF, allowing for intuitive interpretation.

**Fig. 2.**
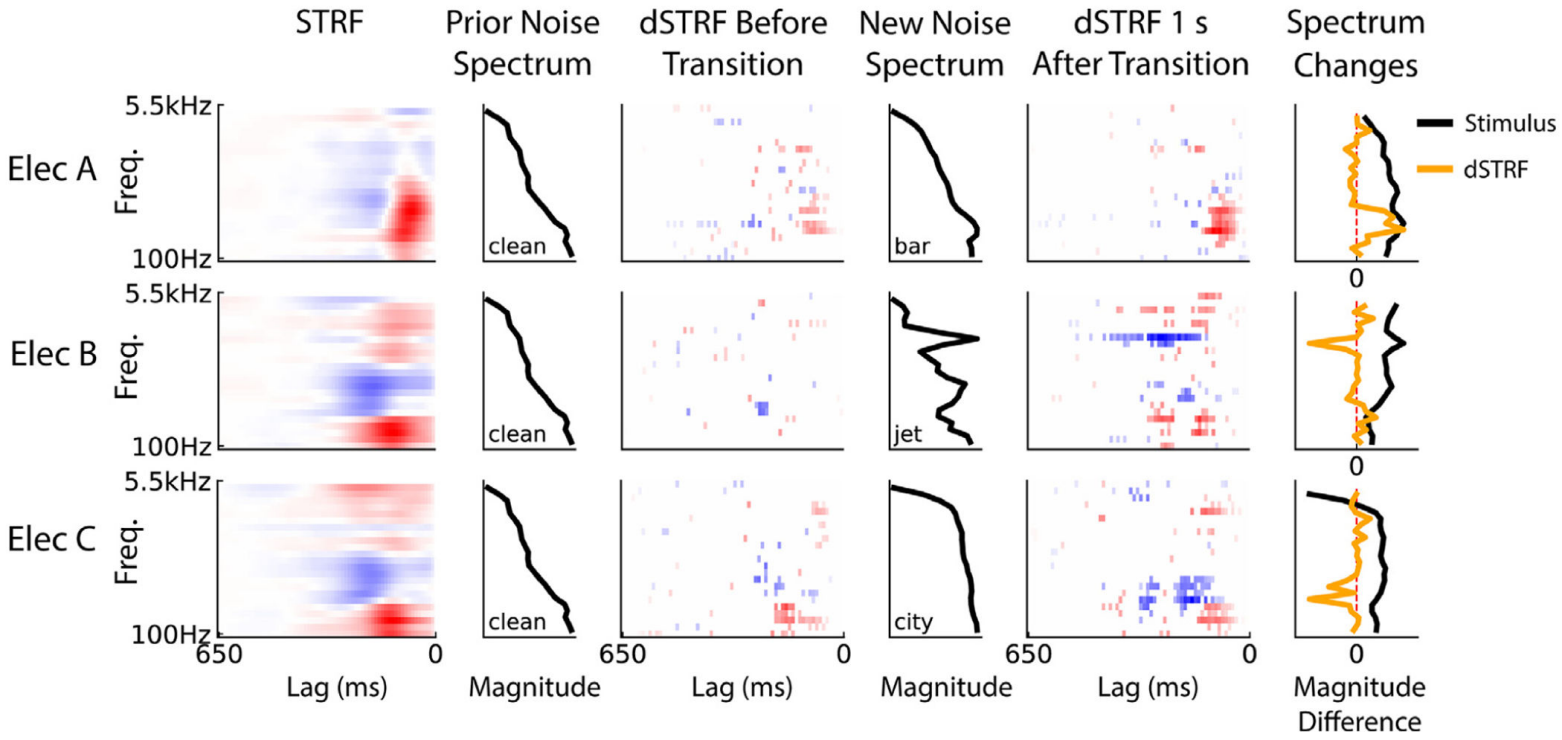
Representative dSTRF frames. dSTRF frames from four electrode models responding to a noise transition: clean-to-bar, clean-to-jet, and clean-to-city, in order from top to bottom. For each electrode, its linear STRF is shown on the left. Then, the spectrum of the noise before the transition is plotted, followed by the dSTRF frame immediately before the change (where the leading lag is a single step before the new noise onset), the spectrum of the new noise after this transition, and the dSTRF 1 s after the noise change. The dSTRF at time *T* seconds relative to the transition is derived by inputting the stimulus spectrogram from time *T*-0.65 to *T* to the DNN model. The rightmost column shows the change in the stimulus spectrum (the difference between the new and old noise spectrums) and the change (from before to 1s after) in the lag-averaged dSTRFs for the given noise transition. Each spectrum magnitude difference is rescaled to have unit maximum absolute value so that they are visually comparable.

We first visualized the dSTRFs of the models to understand how different neural sites alter their filtering after noise transitions. Fig. 2 shows the dSTRFs of several different electrodes as they undergo different types of transitions between clean and noisy backgrounds. The dSTRFs appear sparse in comparison to the STRFs shown in the left column due to the masking method used to keep only significant portions of the dSTRF (see Materials and methods). In general, we observed electrodes change both their gain and shape in response to new noise. For example, electrode A changes its gain in response to transitioning from clean to bar noise, especially increasing the gain of the excitatory region of its receptive field in the peak frequency range of both clean speech and bar noise. Electrode B, during a transition from clean to jet noise, develops a new excitatory region around this same speech spectrum as well as a new inhibitory region of its receptive field at the frequency of the jet noise which results in selective inhibition of the jet noise compared to speech. This is seen in the rightmost column where the large negative change in the dSTRF matches the new increase in high frequency content in the stimulus spectrum. Electrode C illustrates a combination of gain and shape changes following the noise transition, developing a large inhibitory region in its receptive field and changing the size of the excitatory region. The changes exhibited by these dSTRFs have consequences for the neural encoding of speech in noise by adaptively filtering out the new noise content, as we further quantify next.

### 2.3. DNN models exhibit adaptive gain control to account for noise changes

**Fig. 3.**
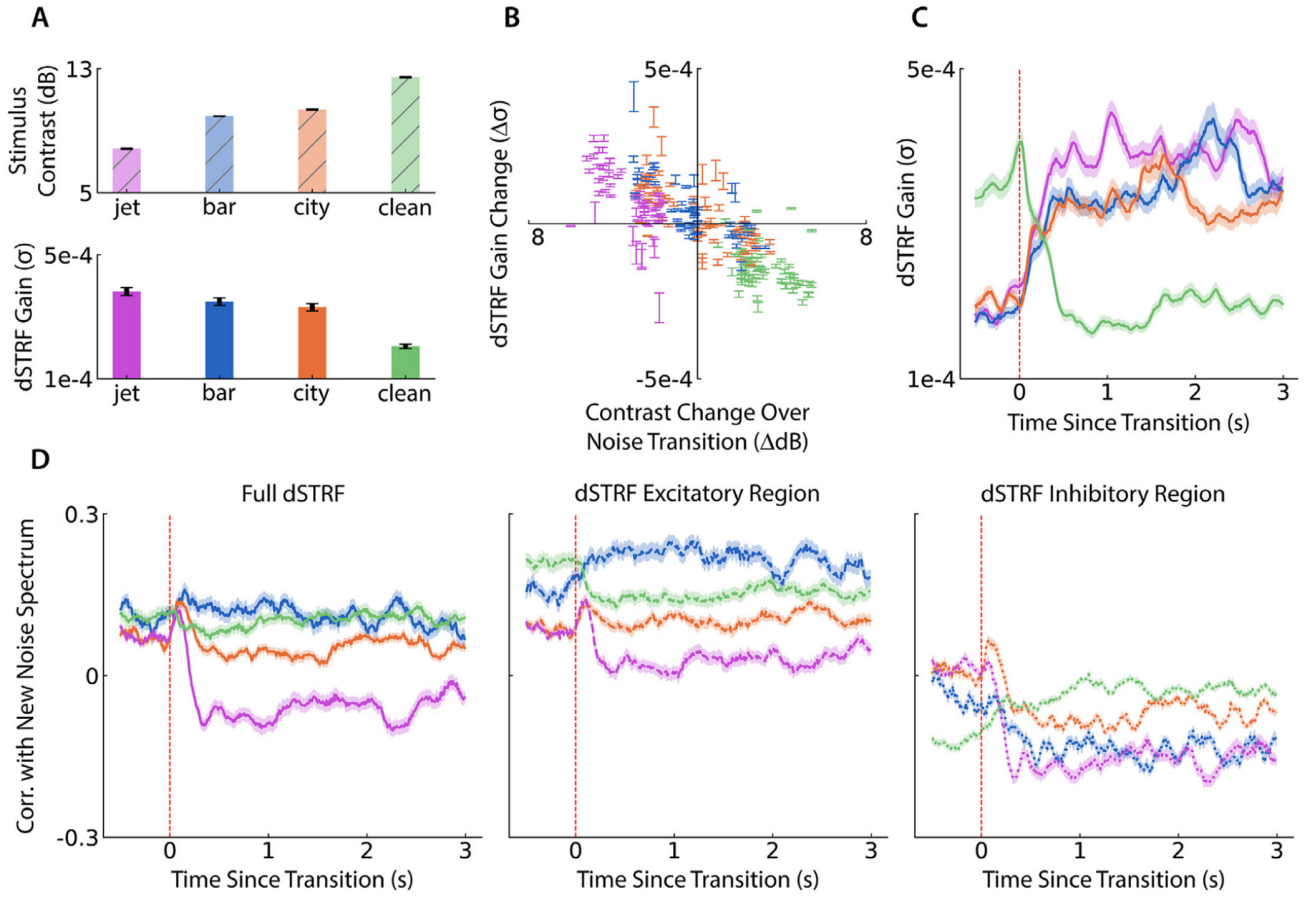
dSTRF gain control and spectro-temporal filter changes around noise changes. (A) (Top) Mean stimulus contrast across all 3/6 s segments of each noise condition type. (Bottom) Average dSTRF gain in each stimulus segment for each noise condition type, illustrating an inverse relationship with stimulus contrast. Bar heights and error bars indicate average and standard error over electrodes. (B) Change in dSTRF gain as a function of stimulus contrast change over a noise transition. Each point shows standard error bars over all electrodes as they undergo a given transition, colored by the noise type after the transition. (C) dSTRF gain over all electrodes over the time course of a transition, restricted to transitions from clean to noise or noise to clean (not noise to noise) in order to ensure a consistent baseline gain value across different to-noise transition types. Any further differences in pre-transition baseline values are attributable to the variability of the clean speech stimuli before the transition. (D) Noise filtering by dSTRFs, measured by the correlation between the lag-averaged dSTRF and the noise spectrum after a transition, averaged over electrodes and transitions but restricted to transitions from clean to noise or noise to clean (not noise to noise). Shaded regions indicate standard error over electrodes. Left plot shows dSTRF correlation with the spectrum of the new noise (or clean speech in the case of noise-to-clean transitions) after the transition, middle plot shows the correlation of only the excitatory region of the dSTRF, and right plot shows the same for only the inhibitory region.

Since some dSTRFs appear to change their gain after noise changes, we investigated whether the DNN model showed evidence of adaptive gain control, whereby neurons maintain a consistent level of activity by adjusting their gain up or down to account for decreases or increases in spectro-temporal contrast in the input stimulus (Cooke et al., 2018; Rabinowitz et al., 2011). We computed the spectro-temporal contrast of the stimulus in each 3/6 s stimulus window, as well as the average gain of each electrode’s dSTRF in each window. Fig. 3A shows average stimulus contrast and dSTRF gain in each of the four noise conditions, showing opposite trends of stimulus contrast and dSTRF gain. Furthermore, around each noise transition in the stimulus we calculated the change in noise contrast and the change in dSTRF gain for each electrode. In Fig. 3B we plot each of these pairs with error bars giving an estimate of the distribution over all electrodes’ gain changes for that noise transition. The negative correlation (Pearson *r* = −0.78, *p* < 0.001) provides further evidence that the DNN models increase or decrease their gain to account for a decrease or increase in stimulus contrast, respectively, and this pattern is consistent across speech responsive electrodes. Finally, to visualize the temporal dynamics of this gain change, we plot the dSTRF gain over transitions in Fig. 3C. To maintain a comparable baseline level for gain changes, we restrict the transitions to those from clean to noise, or from noisy to clean, excluding noise-to-noise transitions. Averaged over electrodes, the gain quickly stabilizes after each type of noise change. These gain change findings indicate that the DNN models use adaptive gain control when reacting to a background noise change, a mechanism that enables them to maintain consistent response levels when the speech content remains consistent but noise conditions vary.

### 2.4. DNN models change receptive field shape to remove new noise

As suggested by electrode B in Fig. 2, which developed a large inhibitory region of its dSTRF upon a transition to jet noise at the frequency where the jet noise had most of its energy, we hypothesized that the DNNs may change their receptive field shape to suppress the noise spectrum. To test this hypothesis, we computed the correlation between the lag-averaged dSTRF at a given time point with the spectrum of the new background noise (or the average clean speech spectrum in the case of transitions to clean speech). Fig. 3D (left) shows these correlations averaged over transitions and electrodes for each transition type. The correlation of the dSTRF with jet noise drops after a transition to jet noise (*p* < 0.001, subject-controlled paired *t*-test between correlations at transition point and 1 s later). This change is not consistent across all types of changes to new noise cases, since the correlation change also drops for city noise (subject-controlled paired *t*-test, *p* < 0.001) but not for bar noise or clean background (subject-controlled paired *t*-test, *p >* 0.05). To determine if noise filtering is a property of the excitatory or inhibitory regions of the dSTRFs specifically, we computed the same correlations using only the non-negative or non-positive regions of the dSTRFs, respectively, as plotted in Fig. 3 D (middle and right). The excitatory region’s behavior is slightly different, since both the bar and city noise correlations increase (subject-controlled paired *t*-test, *p* < 0.001 and *p* < 0.01, respectively), indicating that they respond even more to the noise. On the other hand, the inhibitory region’s correlation drops for all three to-noise transitions, becoming more negative, while the correlation increases for transitions from noise to clean (subject-controlled paired *t*-test, all *p* < 0.001). For transitions to noise, this indicates that the inhibitory region filters out the noise more strongly than before the transition. In the case of a transition to clean speech, this shows that the inhibitory region gets rid of some of its suppression in spectral areas that are prevalent in speech. Taken together, these suggest that the inhibitory region more consistently steers itself away from the spectrum of the new noise and may be responsible for a significant amount of the model’s ability to filter out a new noise.

### 2.5. Gain and spectro-temporal changes predict model improvement over linear STRF

**Fig. 4.**
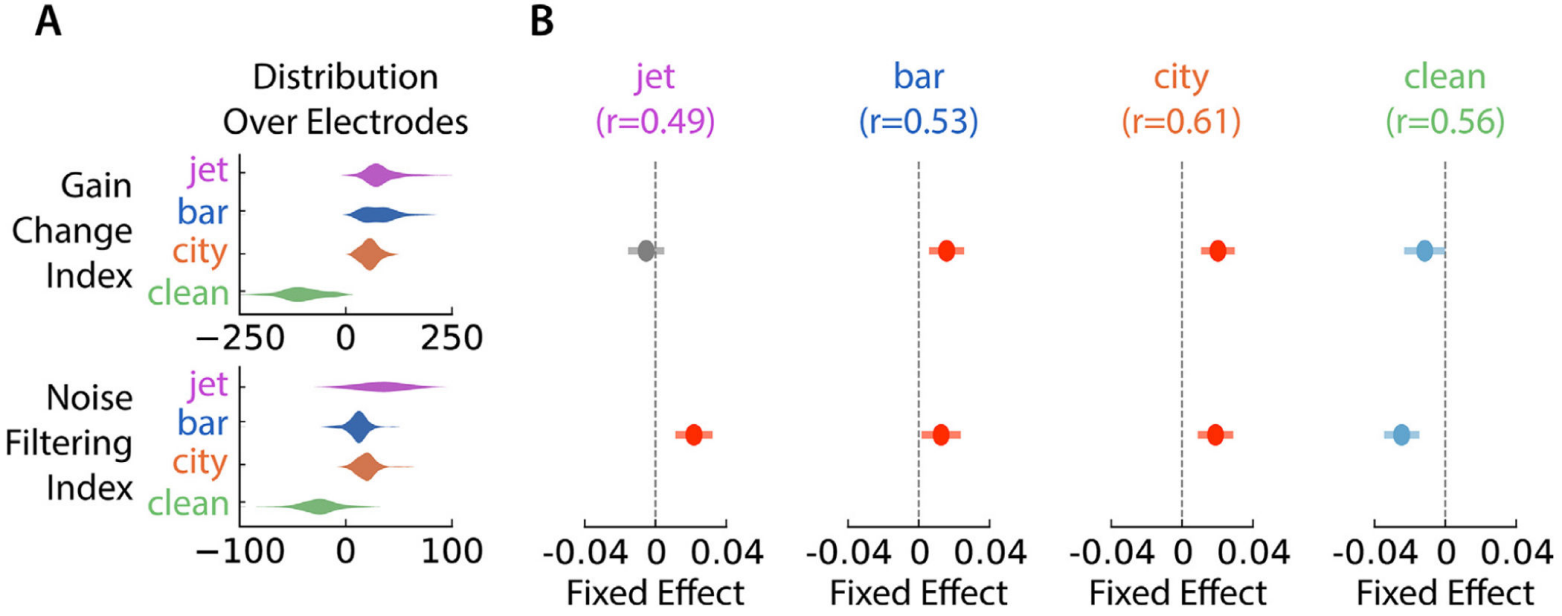
Fixed effects of gain and noise filtering indices on DNN performance improvement. (A) Distribution of gain change and noise filtering indices over electrodes within each background noise class. (B) Fixed effect is shown with confidence intervals from a linear mixed effects model (with subject label as the random effect) predicting an electrode’s DNN correlation improvement over a STRF from gain change and noise filtering indices for each type of noise transition, along with the Pearson correlation to measure the model’s prediction strength in a given noise condition. Red and blue indicate positive and negative effects, respectively. Gray effects were not statistically significant.

To verify that these gain and spectro-temporal change properties had a significant impact on the DNN’s ability to outperform a linear STRF in this adaptation task, we sought to predict the DNN’s correlation improvement using measurements of the gain and spectro-temporal change of each electrode. We used a gain change index to quantify an electrode’s gain change for each of the noise transition types, with the sign of the index indicating the direction of the gain change and the magnitude indicating the size of the gain change. A similar noise filtering index was used to capture the change in the noise spectrum correlation, with a positive index indicating that the dSTRF steered away from the noise spectrum. Since the dSTRF’s inhibitory region exhibited the most significant noise filtering, we used the inhibitory region’s correlation with the noise spectrum to compute the noise filtering index. Both indices were the test statistic from a paired *t* -test between the relevant time-course values in the half second before a transition and a half second starting 650 ms after a transition, with the time-course being the gain around a transition type and the correlation with the new noise around a transition type for each index, respectively. The distribution of these indices over electrodes for each noise condition are plotted in Fig. 4 A, showing a diversity of indices across electrodes, but also that transitions to clean tend to have negative gain change and noise filtering indices, while transitions to noise tend to have positive indices. We hypothesized that gain and noise filtering shape changes would be used by the models in different ways to adapt to different types of noise. Therefore, from these indices within each noise condition, we fit a linear mixed effects model to predict an electrode’s correlation improvement over the linear STRF from its indices in each noise condition. Each model used subject identity as a random effect to control for the impact of varying electrode coverage by subject. Fig. 4B plots the fixed effects from these models capturing the significance of each feature for each noise type. The fixed effect plots show that more positive gain changes in the to-bar and to-city noise transitions, along with greater noise filtering in the to-city, to-bar, and to-jet transitions predicted greater improvement in modeling neural adaptation response patterns. In transitions to clean speech, more negative gain changes and more negative noise filtering, meaning steering toward the spectrum of the speech instead of away from it, predicted better improvement. These findings provide evidence that these nonlinear properties of the DNN enable the adaptive noise suppression in the auditory cortex that the model is capturing.

### 2.6. Noise filtering reveals distinct noise suppression methods along processing pathway

**Fig. 5.**
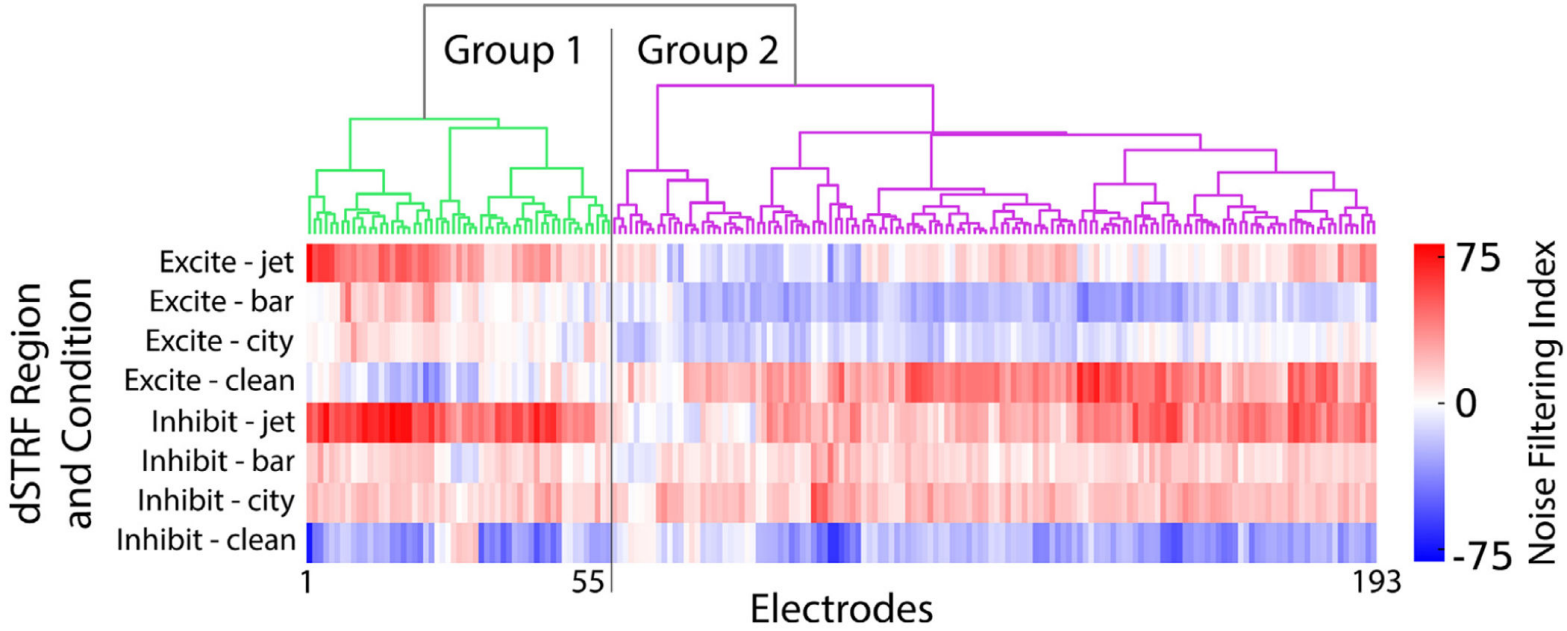
Electrode clustering from noise filtering indices. Hierarchical clustering of electrodes based on noise filtering indices of excitatory and inhibitory regions to each type of noise transition, grouped into two main clusters. Bottom displays the noise filtering indices for each dSTRF region and noise condition.

While the previous plots of dSTRF correlation with noise spectrum suggest that the dSTRF’s inhibitory region is primarily responsible for noise filtering when averaging over all electrodes, we also investigated whether this held true across all electrodes. We computed the same noise filtering index for the dSTRF’s excitatory region in each noise condition, to add to those from its inhibitory region. This resulted in eight indices for each electrode. We then performed hierarchical clustering (minimum variance algorithm, Euclidean distance) over these eight features, and two main groups of electrodes emerged, shown in Fig. 5. While nearly all electrodes exhibit positive noise filtering indices for the three clean-to-noise transitions in their inhibitory regions, a subset of electrodes (group 1) also displays this trend in their excitatory regions, whereas the other subset (group 2) displays mostly negative excitatory noise filter indices for the bar and city transitions. This means that group 1 electrodes use both their excitatory and inhibitory receptive fields to suppress new noise conditions, not just the inhibitory regions, potentially altering their noise suppression abilities.

**Fig. 6.**
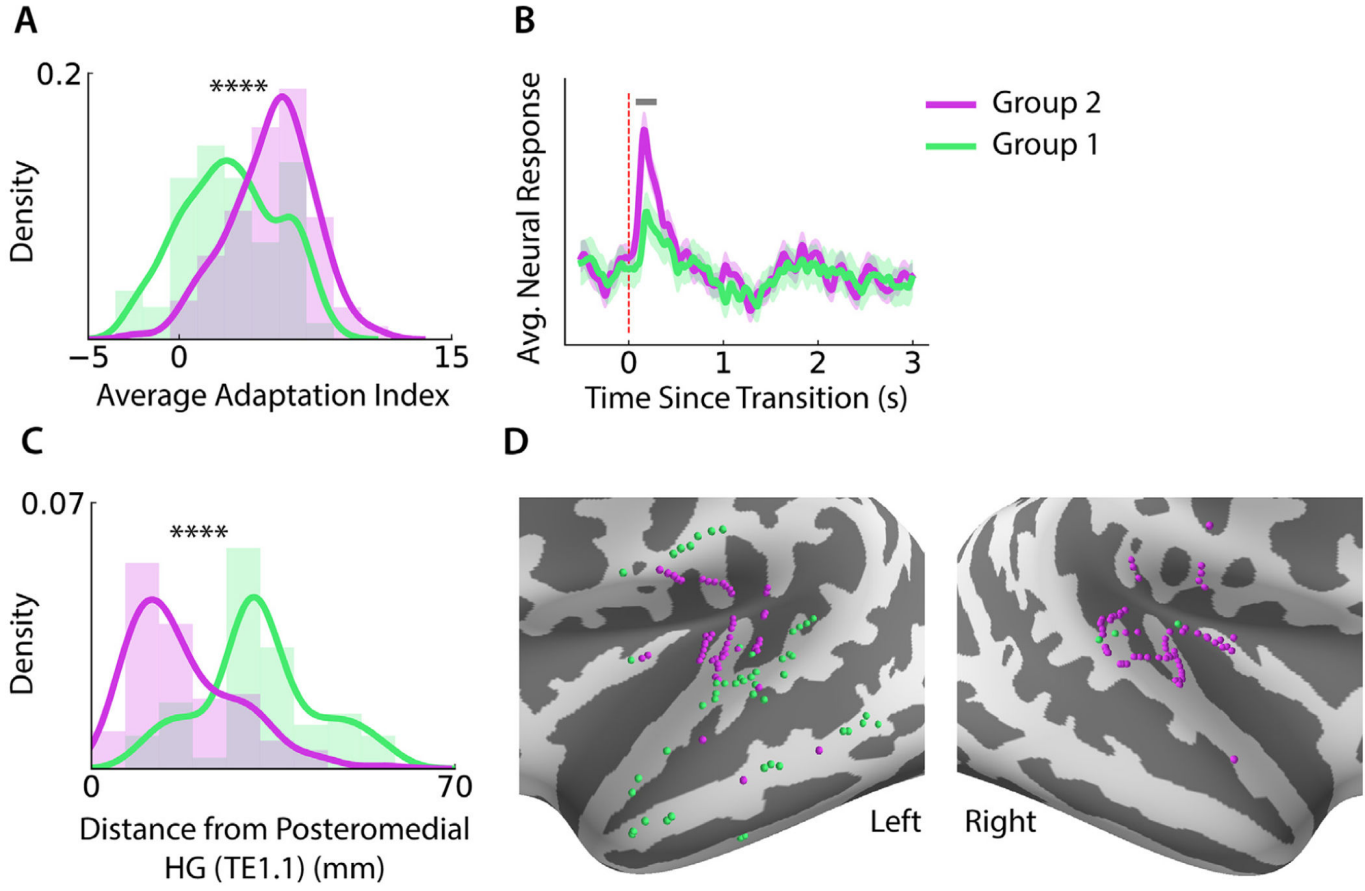
Differences between clustered neural site groups. (A) Histogram and kernel density estimate over electrodes of average adaptation index across all noise transition types, with group 2 electrodes exhibiting significantly higher adaptation indices, with stars indicating significance level. (B) Average neural response to a change in background noise by each group of electrodes. Gray line at the top indicates the temporal region where the responses are significantly different (Wilcoxon ranksum test, *p* < 0.05). Responses from all electrodes are normalized to 0 on average during baseline activity (2–3 s after transition) to ensure the ranksum test compares only the transient responses. Shaded region indicates standard error over electrodes. (C) Histogram and kernel density estimate of electrode distances from posteromedial HG (TE1.1), with group 2 electrodes located significantly closer than group 1 electrodes, with stars indicating significance level. (D) Surface-mapped electrode locations on the inflated FreeSurfer average brain in both left and right lateral views. Most group 2 electrodes are clustered tightly near primary auditory cortex, especially in the left hemisphere, while group 1 electrodes are much more prevalent in nonprimary regions of the left hemisphere.

To understand the effect of this adaptation difference and to confirm that this finding was truly indicative of neural site properties and not simply caused by the models randomly learning one of two potential noise filtering methods, we looked for other differences between the two groups of neural sites. The adaptation index (Khalighinejad et al., 2019) quantifies the magnitude of the transient deviation and subsequent return to baseline immediately following a noise change, with a larger index indicating a larger deviation and return. We compared the average adaptation indices of the electrodes in each group, whose distributions are plotted in Fig. 6 A, and found that group 2 had significantly higher adaptation indices than group 1 (Wilcoxon ranksum test, *p* < 0.001). This suggests that neural sites in group 2 exhibit larger transient responses around noise transitions. This was confirmed by comparing the average neural response to a noise change for each group, as seen in Fig. 6B, where we show that the transient response by group 2 electrodes is significantly higher from 110 ms to 260 ms after the transition (Wilcoxon ranksum test, *p* < 0.05). Next, we examined whether the two groups of neural sites corresponded to different stages of the auditory processing pathway. As a metric for proximity to primary auditory cortex (upstream processing), we computed the distance of each electrode from posteromedial HG (TE1.1) (Baumann et al., 2013; Norman-Haignere and McDermott, 2018). We found that group 1 electrodes are significantly farther than the group 2 electrodes (Wilcoxon ranksum test, *p* < 0.001), shown in Fig. 6C. We confirmed this finding visually by plotting the surface-mapped electrode locations on the average FreeSurfer brain (Fischl et al., 2004), shown in Fig. 6D for the left and right hemispheres. The plots illustrate a clear anatomical division where group 2 electrodes are clustered near primary auditory cortex and group 1 electrodes are spread throughout nonprimary areas, including STG and MTG in the left hemisphere. All together, these findings indicate that the different noise suppression methods used by each group of neural sites influence differences in neural response patterns and adaptation between the groups. The anatomical separation between groups suggests that there are differences in noise filtering mechanisms between primary and nonprimary auditory cortical regions.

## 3. Discussion and conclusion

We used DNNs as a model for the nonlinear adaptation of auditory cortex to changing background noise. We found that DNNs can model the dynamic response patterns seen in auditory cortex, and they significantly outperform the linear STRF and STP models, especially in the period immediately after noise changes during neural adaptation (Khalighinejad et al., 2019). This indicates that the DNNs were not simply better at modeling neural dynamics in steady noise conditions but were also significantly better at modeling the dynamics during the period of noise adaptation. The architecture we used for the DNN models was a CNN with a receptive field of the past 650 ms. Prior research has shown that extracting an auditory object from a temporally dynamic background requires integration over time (Chait et al., 2005; Teki et al., 2011). Although a recurrent network architecture may be naturally suited for integrating temporal information, as may be useful for extracting speech from dynamic noise, our model’s results indicate that a finite-length window is sufficient to reproduce cortical response adaptation to background noise for the noise classes we examined. This supports the choice of a CNN to model the dynamics of neural adaptation.

Despite their nonlinearity, the DNN models we trained were still highly interpretable through their dSTRFs, a key finding which has recently enabled their use as a powerful yet transparent encoding model (Keshishian et al., 2020). Although training a linear STRF model within each noise condition separately might allow for analysis of the basic receptive field changes between the conditions, it suffers from training with a fraction of the total dataset and from being unable to analyze the rapid temporal changes that occur to the receptive field within individual noise windows, and the DNN model allows us to analyze this. Our inspection of these dSTRFs yielded several new insights into the computations which may underlie neural adaptation in auditory cortex. While nonlinear mechanisms such as gain normalization have been theorized to underly neural adaptation to changing background noise (Khalighinejad et al., 2019; Mesgarani et al., 2014; Rabinowitz et al., 2013; Willmore et al., 2014), we demonstrated that models trained to mimic auditory cortical response patterns indeed utilize similar mechanisms when presented with noise changes. The electrode dSTRFs shown in Fig. 2 demonstrate nonlinearities including gain changes, spectro-temporal changes, and combinations of the two, extending previous findings by illustrating the precise dynamics of the filter changes that occur in human auditory cortex in response to changing background noise.

We first investigated the gain changes to see if the model used adaptive gain control to adjust to noise changes. It has been shown that auditory neurons adjust their firing rates to account for stimulus statistics (Dean et al., 2005). One such adjustment is through contrast gain control, a well-studied mechanism displayed by neurons in the cortex and subcortical regions whereby neurons decrease or increase their gain when the spectro-temporal contrast of the auditory stimulus is high or low, respectively (Cooke et al., 2018; Lohse et al., 2020; Rabinowitz et al., 2011; Robinson and McAlpine, 2009). However, prior work on adaptive gain control has investigated its neurophysiological responses in animal models and with simple stimuli, such as mouse auditory cortex. Given the specialization of the human auditory cortex for speech processing (Belin et al., 2000), less is known about how gain control operates in human auditory cortex during naturalistic speech listening. We showed that the models do exhibit adaptive gain control by reducing their gain when entering a new noise condition with higher contrast and increasing their gain when entering a noise condition with lower contrast. This effect was highly consistent across areas and noise transition types. The existence of gain control in our DNN models constitutes an important result since the computations they learn are entirely data-driven, in contrast to those in previous work which assumed a specific model and investigated gain control and noise-robust encoding (Espejo et al., 2019; Mesgarani et al., 2014; Pennington and David, 2020; Rabinowitz et al., 2012). These results advance our understanding of adaptive gain control by demonstrating how it arises in human auditory cortex during real-world rapid noise changes.

We next examined the spectro-temporal changes that the dSTRFs undergo in response to noise changes. Since auditory cortical responses have been shown to selectively encode vocalizations over background noises in constant or changing background conditions (Khalighinejad et al., 2019; Mesgarani et al., 2014; Moore et al., 2013; Narayan et al., 2007; Rabinowitz et al., 2013; Schneider and Woolley, 2013), we hypothesized that the spectro-temporal change during the adaptation period constituted the model attempting to find a new filter that would remove the new background and keep the speech signal. We confirmed this by finding that the dSTRF’s inhibitory region changes to become anticorrelated with the new noise spectrum. Prior work has shown that A1 neuron STRFs exhibit different patterns which maximize target detection when animals are engaged in sound discrimination compared to baseline (Atiani et al., 2009; Fritz et al., 2003). While the reported changes in those studies were induced by a change in the behavioral state of the animal, our study shows the utility of similar computations when the task remains the same, but the background changes, requiring a new computation for maintaining the optimal representation that supports speech perception. As such, we can interpret the rapid spectro-temporal changes in our models as neural sites adapting to a new sensory context (David, 2018), where the behavioral goal of maintaining enhanced responses to the target stimuli (speech) in the presence of a realistic background noise largely remained constant. The continued focus on speech content may still be related to top-down at-tentional modulation of ascending auditory processing. A future extension of this work may also consider behavioral context, such as task engagement or attention changes (Atiani et al., 2014; Fritz et al., 2003, 2005; Fritz et al., 2007; Mesgarani and Chang, 2012) and perceptual learning (Ohl et al., 2001; Ohl and Scheich, 1997; Polley et al., 2006), as an input to the neural network model which could learn a joint nonlinear encoding of stimulus and behavioral context. Our results also go beyond the previous characterization of receptive field plasticity (Atiani et al., 2009; Fritz et al., 2003, 2005) by showing that the dynamic changes in the inhibitory regions of the receptive field may be crucial to real-world noise adaptation. This provides new evidence of the precise computations that the human auditory cortex may use to suppress background noise in a dynamic acoustic environment.

After characterizing the model’s gain and spectro-temporal change abilities, we confirmed that they played a significant role in the DNN’s modeling ability by using each DNN’s nonlinear properties to predict the model’s correlation improvement over a STRF and adaptive models such as STP. These models revealed that gain change was most important in transitions to bar noise, city noise, and clean speech, but not for jet noise. On the other hand, noise filtering was important for all types of transitions but did not have as big of a fixed effect in bar transitions as gain changes. These differences can be explained by the more similar spectrum of bar noise to that of speech, while jet noise is the most different from speech. So, a change in spectro-temporal receptive field shape which removes jet noise can benefit noise suppression without degrading speech responses, but any receptive field shape change which removes bar noise will degrade speech responses as well, given their similar spectro-temporal profile (Chi et al., 2005). Thus, when the environment changes from clean speech to bar noise in the background, auditory cortical sites might need to rely more on gain changes than spectro-temporal changes to continue encoding speech content properly. Prior research has identified energetic and informational masking, when a distractor or noise signal partially masks a target signal through overlapping spectro-temporal content, as an important aspect of noisy tone detection and speech comprehension (Brungart et al., 2001; Kidd et al., 2002). When testing tone detection in noise, it has been shown that behavioral detection is worse when maskers overlap the signal more (Neff and Green, 1987; Oh and Lutfi, 1998; Woods et al., 1994). Our results provide a neural correlate of this behavioral finding and show that, for naturalistic sounds, changes to receptive field gain and shapes operate independently depending on noise spectra to enable auditory cortical regions to quickly adapt to new masking conditions.

We further identified two distinct groups of neural sites based on noise filtering in their receptive fields. A subset of the models steered both the excitatory and inhibitory regions of their receptive fields away from new noise spectrums, while other sites only used changes in their inhibitory receptive fields to reduce noise responses. These differences also highlighted interesting neural and anatomical properties of these populations. We found that the neural sites in these groups had very different transient responses to noise changes, as measured by the adaptation index. The group of sites whose models also used their excitatory regions to filter out new noise had lower adaptation indices and correspondingly smaller transient responses to noise transitions, suggesting that these sites’ models utilize both excitatory and inhibitory adaptive changes in order to reduce the transient response to a noise change. Additionally, the neural sites in this group were located throughout non-primary auditory cortex and further cortical regions, while the other group of neural sites was clustered in and around primary auditory cortex. Prior work has demonstrated differences in STRF tuning changes by neural sites with best frequencies near or far from a target tone when task difficulty is altered (Atiani et al., 2009). However, auditory cortical neurons can be described by several different tuning dimensions beyond frequency, such as temporal and spectral modulations (Walker et al., 2011), and with complex stimuli like speech, there is likely more involved than just best frequency tuning. Our findings unveil a portion of this added complexity by identifying differences in the nonlinear computations being performed to filter out background noise as an acoustic representation moves down the auditory processing pathway. It has been shown that noise-robustness increases down the auditory pathway (Las et al., 2005; Rabinowitz et al., 2013; Schneider and Woolley, 2013), and more specifically nonprimary auditory cortical representations are more robust to real-world noise than primary auditory cortex (Kell and McDermott, 2019; Kell and McDermott, 2017), and our models provide a potential computational explanation.

Previous work has shown that spectro-temporal tuning and response selectivity in higher order auditory cortex is modulated by task demands and attention (Atiani et al., 2014; Fritz et al., 2007; Petkov et al., 2004; Puvvada and Simon, 2017), so the DNN’s anatomically-grouped noise filtering properties could be an indication that the model is mimicking attention-related tuning to the speech stimuli. On the other hand, it was shown that auditory cortical neural responses to changing background noise are not significantly different with and without attention (Khalighinejad et al., 2019). Thus, while the function of the model’s noise suppressive tuning changes is apparent, it is difficult to determine its origin. Comparing the same sort of data-driven models which are instead trained to predict responses from subjects with and without attention to the task may illuminate greater differences in the response patterns and the underlying computations that drive them than an analysis of the responses alone.

Overall, we used DNN models to reveal multiple nonlinear computations that can explain and predict neural adaptation to changing background noises in human auditory cortex. Our inspection of these models showed that they reproduce cortical computations which have been previously identified and propose potential new mechanisms towards fully accounting for the underlying computations that give rise to the invariant cortical representation of speech and robust speech perception in adverse acoustic environments.

## 4. Materials and methods

### 4.1. Human subject intracranial recording

Six subjects participated in the study as they were undergoing clinical evaluation for drug-resistant epilepsy at North Shore University Hospital. Electrodes were implanted according to the clinical goal of identifying epileptogenic foci for later surgical removal, and any electrodes which were identified by an epileptologist as showing any sign of epileptiform discharges were removed from the pool of electrodes for analysis here. All iEEG recordings were manually inspected to ensure they were free of interictal spikes. All subjects gave written informed consent to participate in this research before implantation of electrodes, and the research protocol was approved by the Feinstein Institute for Medical Research institutional review board. Subjects listened to a total of approximately 20 min of stimuli (described below) while recordings were taken. All recordings were acquired at 3 kHz sampling rate with a data acquisition module (Tucker-Davis Technologies, Alachua, FL, USA). The envelope of the high-gamma response was extracted with the Hilbert transform (Edwards et al., 2009). This was then downsampled to 100 Hz. To identify responsive electrodes, we performed a *t*-test between each electrode’s response time-point-wise over 0.5 to 0 s immediately preceding the first speech onset compared to 0 to 0.5 s immediately following the first speech onset. Across all subjects, a total of 193 electrodes were identified for analysis, with each subject contributing at least 23 and no more than 41. Electrode responses were normalized based on the mean and variance of the response in a 2 min silent interval taken before the task.

### 4.2. Subject-controlled statistical tests

Since the electrodes come from 6 underlying subjects, we modified our statistical tests to account for this grouping factor, when applicable. For one-sample and relative *t*-tests which tested the distribution of all electrodes, we used a subject-controlled *t*-test under a linear model framework. To do this, we added one-hot-encoded subject identity features to the typical design matrix used to compute the *t*-test statistics and p-values, thus removing the potential effect of subject identity from distribution shifts.

### 4.3. Acoustic stimulus and model input spectrogram

The stimuli used in this study consisted of approximately 20 min of speech from 2 male and 2 female voice actors reading short stories, which was added to background noise that changed between four main classes: bar noise, city noise, jet noise, and a clean (empty) background. Noises from the same class were unique sound segments added at a 6 dB signal-to-noise ratio, a level chosen to ensure speech intelligibility (Bradley et al., 1999). These noise classes contain a diversity of spectra which allows for the analysis of adaptation to noises which are both very similar to (bar) and different from (jet) speech. Stimuli were presented from a Bose SoundLink Mini 2 speaker placed in front of the participant. The volume was adjusted to a comfortable listening level for the subject. The stimuli were segmented into 18 blocks of approximately equal length, and after each block, the subject was asked to repeat the last sentence they heard to check their attentiveness.

We transformed the acoustic stimuli into 23-channel Mel spectrograms at 100 Hz for input into both the DNN and STRF models. The Mel spectrogram was chosen because it produced consistently smooth STRFs for all electrodes, compared to other time-frequency representations, and the small number of frequency bands restricted the number of channels to enable a more manageable and interpretable analysis of dSTRFs.

### 4.4. Model training

STRF models were trained with normalized reverse correlation using STRFLab (Theunissen et al., 2001). We set the tolerance and sparseness parameters using cross-validation, with tolerance values swept between 0.01 and 0.1 and sparseness between 0 and 2.

The DNN models were 5-layer 1D convolutional neural network (CNN) models with ReLU activations and a final linear projection layer. All layers used 128 kernels with a kernel size of 5, a stride of 1, and no padding. Only the final linear projection layer had a bias. The first two convolutional layers had a dilation of 1, and the remaining three layers had dilations of 2, 4, and 8, respectively. This produced a model with a receptive field of 65 samples, or 650 ms. All layers were shared across all electrodes with the final layer predicting all electrodes’ responses at the same time. The objective function during training was the mean-squared error of the predictions, averaged across electrodes. We used the RAdam optimizer, an exponential learning rate scheduler with a decay rate of 0.996, and weight decay regularization of 0.03. DNN models were trained with PyTorch (Paszke et al., 2019).

STRF and DNN models both had a receptive field of the previous 650 ms of the stimulus spectrogram. All models were trained using a cross-validated jackknifing procedure across the 18 natural division blocks (approximately 1 min each) in the auditory stimulus. Keeping a given division as held-out test data, the remaining 17 divisions were used as the training set for a jackknifing procedure where one division was withheld and a model was trained on the remaining 16 divisions, leading to 17 models being trained for the same test data. To compute the predictions for the held-out test data, the predictions of these 17 models were averaged.

### 4.5. STP model comparison

The STP model consisted of a linear-nonlinear (LN) model, followed by a short-term plasticity module. The LN portion we used was a 650 ms finite impulse response (FIR) filter followed by a double exponential static nonlinearity. The STP portion is parameterized by the twoparameter Tsodyks-Markram model (Espejo et al., 2019; Tsodyks et al., 1998). STP models were fit using the Neural Encoding Model System (David, 2018). Due to the extensive training time for the STP model, a single train-test split was used to compute correlation scores and it was compared to the DNN scores for retrained DNN models for the same train split. The scores for this split were representative of overall scores since the DNN scores were highly correlated with the cross-validated DNN scores used elsewhere in this paper (Pearson *r* = 0.94, *p* < 0.001).

### 4.6. dSTRF calculation

The dSTRF can be computed easily from a neural network with rectified linear unit nodes (ReLU) since these networks implement piecewise linear functions. To compute the dSTRF for a CNN, we begin by converting the CNN into a multilayer perceptron (MLP) (Keshishian et al., 2020), since it is simpler to calculate the dSTRF for an MLP. If the MLP uses ReLU activations and does not contain bias in its intermediate layers, the dSTRF is equivalent to the gradient of the output with respect to the network’s input vector (Nagamine and Mesgarani, 2017), which is defined as follows:

$$dSTRF\left(x_{t}\right)=\frac{\partial {\hat{y}}_{t}}{\partial x_{t}}=\frac{\partial {\hat{y}}_{t}}{\partial z_{t}^{l}}\frac{\partial z_{t}^{l}}{\partial h_{t}^{l-1}}\frac{\partial h_{t}^{l-1}}{\partial z_{t}^{l-1}}\frac{\partial z_{t}^{l-1}}{\partial h_{t}^{l-2}}\cdots \frac{\partial h_{t}^{1}}{\partial z_{t}^{1}}\frac{\partial z_{t}^{1}}{\partial x_{t}}\;\;=\frac{\partial {\hat{y}}_{t}}{\partial z_{t}^{l}}\;W_{l-1}^{l}\frac{\partial h_{t}^{l-1}}{\partial z_{t}^{l-1}}\;W_{l-2}^{l-1}\;\cdots \;\frac{\partial h_{t}^{1}}{\partial z_{t}^{1}}\;W_{Input}^{1}$$

Above, $z_{t}^{l}$ represents the weighted sum of inputs to layer l for the input $x_{t}$, and $h_{t}^{1}$ indicates the output from layer l. The weights from layer l−1 to l is denoted by $W_{l-1}^{l}$. The gradient is simply the product of the gradients of each layer, each of which contain a weight matrix and node activation function. Since the network only uses ReLU activations at the nodes, the gradient of the activations reduces to the following:

$$\frac{\partial h(\cdot )}{\partial z(\cdot )}=\{\begin{array}{ccc} 1 & if & z>0\\ 0 & if & z<0 \end{array}$$

Thus, the product of the activation gradient $\frac{\partial h(\cdot )}{\partial z(\cdot )}$ and the weight matrix $W_{l-1}^{l}$ can be rewritten based on when the output is nonzero, using the indices m and n corresponding to nodes in layers l and l−1, respectively:

$${\hat{W}}_{l-1}^{l}\left(x_{t}\right)[m,n]=\{\begin{array}{l} W_{l-1}^{l}[m,n]\;if\;h_{t}^{l}[m]>0\\ 0\;otherwise \end{array}$$

And therefore, the dSTRF is simply the product of these rewritten weight matrices:

$$dSTRF\left(x_{t}\right)={\hat{W}}_{l-1}^{l}{\hat{W}}_{l-2}^{l-1}\ldots {\hat{W}}_{Input}^{1}$$

Rather than converting each CNN into an MLP and calculating this gradient manually, we used the automatic differentiation functionality of PyTorch (Paszke et al., 2019) to compute the dSTRF directly from the CNN.

In order to produce robust dSTRFs, the dSTRF for the held-out test division was computed by averaging over the 17 dSTRFs of the models trained in the jackknifing procedure. To further remove noise from the dSTRFs due to DNN training stochasticity, an additional sign-consistency filtering was applied so that for a given time-frequency bin at a given time point, if the values did not agree in sign for at least 15 of the 17 trained models, the average was set to zero.

### 4.7. Computing stimulus contrast and dSTRF gain

Stimulus contrast was defined as the standard deviation of all bins in the time-frequency representation of the noisy stimulus within a given 3-or 6 s segment of stimulus. These values were then converted to log-scale to plot in decibels.

To compute gain, dSTRFs were aligned to the start of a new noise and grouped by background noise condition. In order to standardize the baseline levels for dSTRF changes around noise transitions in Fig. 3C, only transitions to a specific type of noise which came from a clean background were analyzed, while transitions to clean background include those coming from any noise type. Gain at a single time point was defined as the standard deviation of the dSTRF lag-frequency filter. The gain of the excitatory region was defined as the standard deviation of the dSTRF filter when all negative bins were set to zero, and the same was done for the gain of the inhibitory region with positive bins set to zero.

### 4.8. Computing dSTRF noise filtering

dSTRFs were aligned to the start of a new noise in the same way as for gain changes, also only including transitions between clean and noisy backgrounds, not noise-to-noise transitions. For each of the 3 noisy backgrounds, the average spectrum was computed using the Mel-spectrogram of the noise audio alone and averaging over time. For the clean background, the average spectrum was computed in the same way using the full task stimulus without any additive noise. To compute the dSTRF’s correlation with one of these spectrums at a given time, the dSTRF lag-frequency filter was averaged over lags and the Pearson correlation between this frequency spectrum and the spectrum of the new noise after a given transition was calculated. For the excitatory-or inhibitory-specific correlations, the average over lags was taken after first zeroing out all negative or positive lag-frequency bins in the dSTRF, respectively.

### 4.9. Gain change and noise filtering indices

To capture the gain change by each dSTRF in a single index, we used the test statistic from a paired *t* -test between the gain values (computed above) 0.5 to 0 s before a given transition and 0.65 to 1.15 s after the transition, the first time-window following the adaptation period. A positive test statistic indicated an increase in the gain values. Rather than measuring the magnitude change from pre-transition to post-adaptation, we used a test statistic from a *t*-test because it favors electrodes which adapt their receptive fields and maintain a consistent new gain with low gain variability. A metric like the average gain change would instead favor the raw magnitude of a gain change without considering the variance around the gain on either side of the transition, which would be more prone to noisy gain fluctuations and would create inherently larger gain change indices around different types of noise changes (clean-to-jet compared to clean-to-bar) simply depending on the stimulus gain change, not on the model’s concerted adaptation to it. Similarly, the noise filtering index was computed with the same *t*-test procedure but using the noise spectrum correlations instead of gain values. Additionally, a positive test statistic indicated the correlation decreased, meaning the dSTRF steered away from the new noise spectrum.

### 4.10. Calculating adaptation indices

The adaptation index (Khalighinejad et al., 2019) for each electrode was computed as the test statistic from a paired *t*-test between the electrode’s neural response 0–0.7 s and 2–2.7 s after a noise transition, with a more positive index signifying a larger drop back to baseline. The mean adaptation index over the 4 noise conditions for each electrode was used as its single average adaptation index.

### 4.11. Electrode localization, distance, and visualization

Electrode positions were mapped to the subject’s brain anatomy by co-registration between pre- and post-implant MRI using iELVis (Groppe et al., 2017), and they were identified on the post-implant CT scan with BioImage Suite (Papademetris et al., 2022). These electrode locations were then mapped to the FreeSurfer average brain (Fischl et al., 2004) and their 3-dimensional Euclidean distance from the centroid of posteromedial HG (TE1.1) (Morosan et al., 2001) in this average brain was computed, since TE1.1 is a common landmark for primary auditory cortex (Baumann et al., 2013; Norman-Haignere et al., 2022; Norman-Haignere and McDermott, 2018). To visualize electrodes, electrode locations were mapped to the average FreeSurfer brain template, subdural electrodes were snapped to the closest point on the surface, and all electrodes were plotted on the inflated brain.

## Supplementary Material

1

## Data Availability

The iEEG data used in this study cannot be made publicly available but can be requested from the author [N.M]. Code for extracting the high-gamma envelope from neural data is available at https://github.com/Naplib/Naplib (Khalighinejad et al., 2017). Code for identifying significant electrodes, performing subject-controlled *t*-tests, and fitting mixed effects models is available as part of the naplib-python package at https://github.com/naplab/naplib-python. The code for DNN training and dSTRF estimation is available on Github (Keshishian et al., 2020).

## Data Availability

Data will be made available on request.
