# Supplementary material for: Deep neural networks effectively model neural adaptation to changing background noise and suggest nonlinear noise filtering methods in auditory cortex
Source: Neuroimage. Author manuscript; Available in PMC 2023 Sep 20. (PMC10510744; doi:10.1016/j.neuroimage.2022.119819)
Supplement: 1 [file NIHMS1867081-supplement-1.docx]

# Deep neural networks effectively model neural adaptation to changing background noise and suggest nonlinear noise filtering methods in auditory cortex

Gavin Mischler ^a,b^, Menoua Keshishian ^a,b^, Stephan Bickel ^c^, Ashesh D. Mehta ^c^, Nima Mesgarani ^a,b,d^

^a^ Mortimer B. Zuckerman Mind Brain Behavior, Columbia University, New York

^b^ Department of Electrical Engineering, Columbia University, New York

^c^ Hofstra Northwell School of Medicine, Manhasset, New York

^d^ Corresponding author, nima@ee.columbia.edu

## Supplemental Material


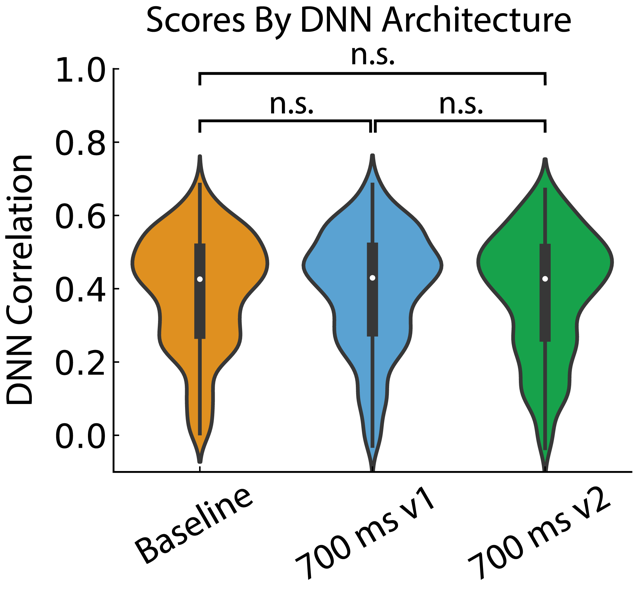


**Fig.** **S1. DNN architecture performance.** Prediction correlations from the baseline 650 ms model on the first test block are compared to those of two other model architectures to illustrate that receptive field size and architecture do not significantly impact model performance. The first model (v1) has a 700 ms receptive field using the same general architecture as the baseline but changing the first two layers to use kernel sizes of 7 and 8, respectively. The second (v2) is a model with kernel sizes of [2, 5, 9, 9, 11] and dilations of [1, 1, 1, 2, 4]. Subject-controlled paired t-tests between all pairs showed no significant differences (p>0.05).


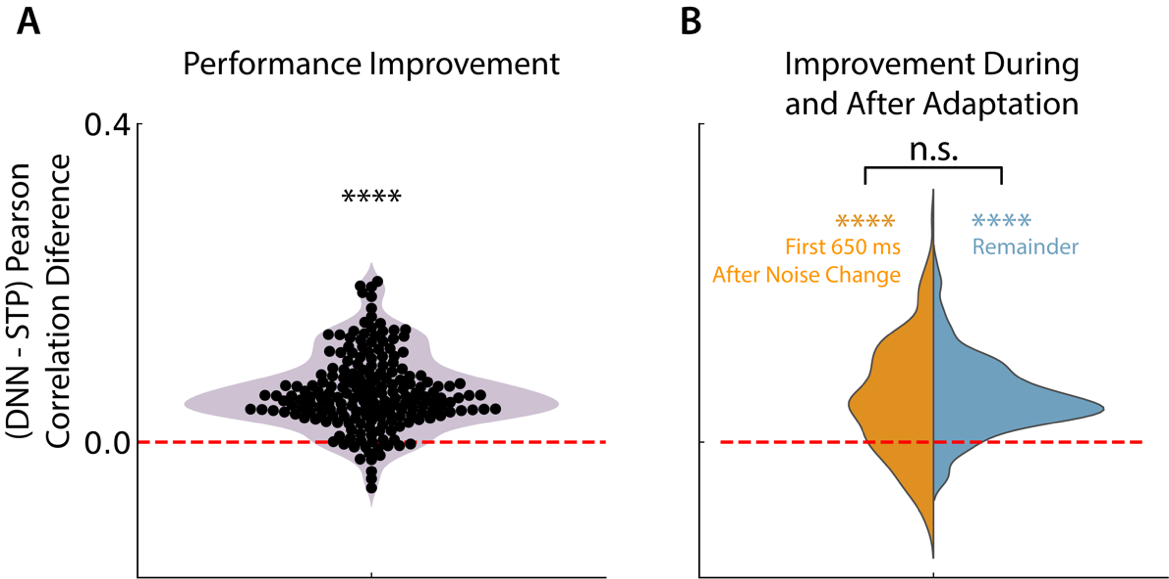


**Fig.** **S2. DNN performance improvement over STP model.** A) Distribution is over all electrodes of DNN model correlation improvement over the STP model for the full task. Stars indicate significance above 0 (t-test). B) Distribution over all electrodes of DNN model correlation improvement over STP computed individually for the periods during and after adaptation. Stars indicate that each correlation improvement distribution is significantly above zero (t-test), though the two distributions are not significantly different from each other (paired t-test).


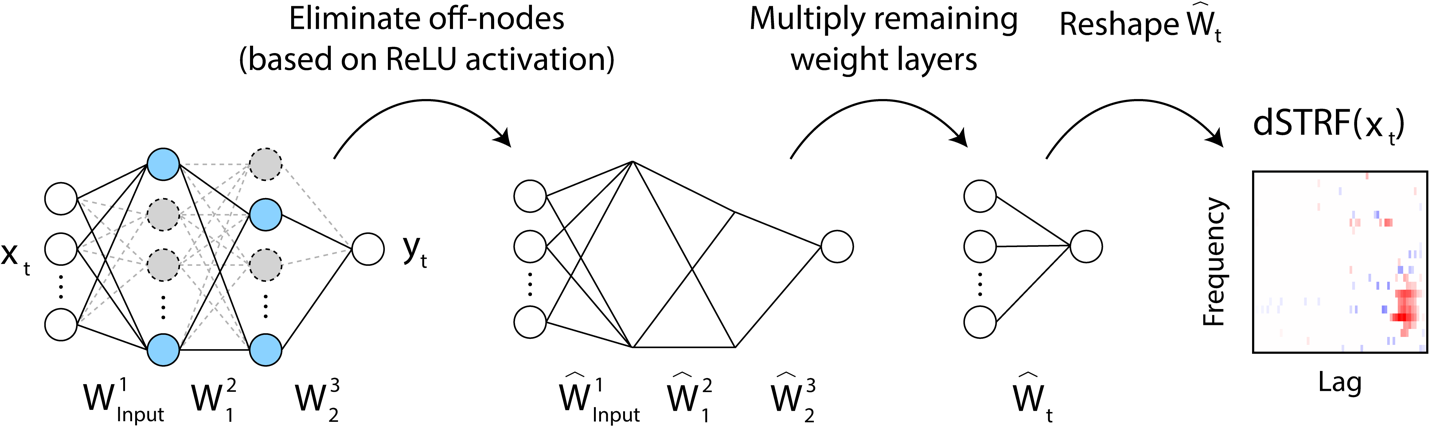


**Fig.** **S3. dSTRF calculation.** An illustration of the steps to calculate the dSTRF from an MLP based on a given stimulus instance. Lines connecting nodes represent weights between those nodes. The stimulus instance $x_{t}$ is the lag-frequency representation in the model’s current receptive field which has been reshaped into a vector to be input into the MLP. For this stimulus instance, blue nodes indicate nodes with positive output after the ReLU activation, while gray nodes are off since their output is zero after ReLU activation. First, nodes whose output is 0 are removed. Then, the remaining weights are multiplied together to produce a single set of equivalent weights which transform the input to the output. Then these weights are reshaped back to the shape of the input to produce a dSTRF which can be visualized like a STRF.
